# 3-(6-Bromo­hex­yl)-1,5-dimethyl-1*H*-1,5-benzodiazepine-2,4(3*H*,5*H*)-dione

**DOI:** 10.1107/S1600536810040122

**Published:** 2010-10-13

**Authors:** Rchida Dardouri, Fouad Ouazzani Chahdi, Natalie Saffon, El Mokhtar Essassi, Seik Weng Ng

**Affiliations:** a Laboratoire de Chimie Organique Hétérocyclique, Pôle de Compétences Pharmacochimie, Université Mohammed V-Agdal, BP 1014 Avenue Ibn Batout, Rabat, Morocco; b Service Commun Rayons-X FR2599, Université Paul Sabatier, Bâtiment 2R1, 118 route de Narbonne, Toulouse, France; c Department of Chemistry, University of Malaya, 50603 Kuala Lumpur, Malaysia

## Abstract

The seven-membered ring in the title compound, C_17_H_23_BrN_2_O_2_, adopts a boat-shaped conformation (with the C atoms of the fused-ring as the stern and the methine C atom as the prow). The bromo­hexyl substituent occupies an equatorial position, with the hexyl chain exhibiting an extended conformation. Weak inter­molecular C—H⋯O hydrogen bonding is present in the crystal structure.

## Related literature

For the crystal structure of 1,5-dimethyl-1,5-benzodiazepin-2,4-dione, see: Mondieig *et al.* (2005 ▶).
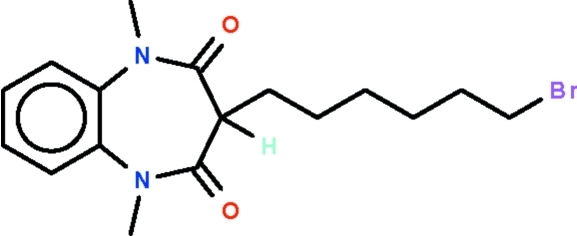


## Experimental

### 

#### Crystal data

C_17_H_23_BrN_2_O_2_
*M* *_r_* = 367.28
Monoclinic, 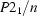

*a* = 7.5214 (1) Å
*b* = 9.3693 (2) Å
*c* = 23.8686 (5) Å
β = 91.750 (1)°
*V* = 1681.24 (6) Å^3^
*Z* = 4
Mo *K*α radiation
μ = 2.45 mm^−1^
*T* = 293 K
0.30 × 0.20 × 0.10 mm

#### Data collection

Bruker X8 APEXII diffractometer
Absorption correction: multi-scan (*SADABS*; Sheldrick, 1996 ▶) *T*_min_ = 0.526, *T*_max_ = 0.791
25590 measured reflections
4897 independent reflections
3478 reflections with *I* > 2σ(*I*)
*R*_int_ = 0.045

#### Refinement

*R*[*F* ^2^ > 2σ(*F* ^2^)] = 0.051
*wR*(*F* ^2^) = 0.153
*S* = 1.01
4897 reflections
201 parameters
H-atom parameters constrained
Δρ_max_ = 1.92 e Å^−3^
Δρ_min_ = −0.68 e Å^−3^

### 

Data collection: *APEX2* (Bruker, 2008 ▶); cell refinement: *SAINT* (Bruker, 2008 ▶); data reduction: *SAINT*; program(s) used to solve structure: *SHELXS97* (Sheldrick, 2008 ▶); program(s) used to refine structure: *SHELXL97* (Sheldrick, 2008 ▶); molecular graphics: *X-SEED* (Barbour, 2001 ▶); software used to prepare material for publication: *publCIF* (Westrip, 2010 ▶).

## Supplementary Material

Crystal structure: contains datablocks global, I. DOI: 10.1107/S1600536810040122/xu5049sup1.cif

Structure factors: contains datablocks I. DOI: 10.1107/S1600536810040122/xu5049Isup2.hkl

Additional supplementary materials:  crystallographic information; 3D view; checkCIF report

## Figures and Tables

**Table 1:** Hydrogen-bond geometry (Å, °)

*D*—H⋯*A*	*D*—H	H⋯*A*	*D*⋯*A*	*D*—H⋯*A*
C7—H7*B*⋯O1^i^	0.96	2.58	3.430 (3)	147
C7—H7*C*⋯O2^ii^	0.96	2.51	3.471 (3)	174
C11—H11*B*⋯O1^ii^	0.96	2.60	3.551 (3)	173

Symmetry codes: (i) 
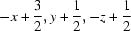
; (ii) 
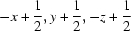
.

**Fig. 1.**
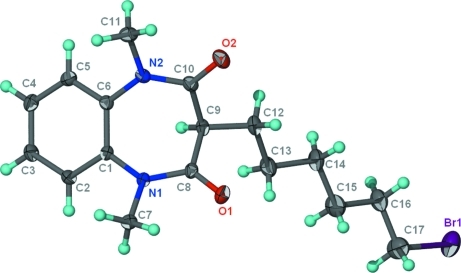
Thermal ellipsoid plot (Barbour, 2001) of C17H23BrN2O2 at the 50% probability level; hydrogen atoms are drawn as arbitrary radius.

## supplementary crystallographic information

### Comment

The methylene part of 1,5-dimethyl-1,5-benzodiazepine-2,4-dione is relatively acidic, and one proton can be abstracted by using potassium *t*-butoxide; the resulting carbanion can undergo a nucleophlilic subsitution with a dibromoalkane to form 3-substituted derivatives. In this study, the compound is reacted with 1,6-dibromohexane the title compound (Scheme I, Fig. 1).

### Experimental

To a solution of the potassium *t*-butoxide (0.42 g, 3.6 mmol) in DMF (15 ml) was added 1,5-dimethyl-1,5-benzodiazepine-2,4-dione (0.50 g, 2.4 mmol) and 1,6-dibromodohexane (0.40 ml, 2.88 mmol). Stirring was continued for 24 h. The reaction was monitored by thin layer chromatography. The mixture was filtered and the solution evaporated to give colorless crystals.

### Refinement

Carbon-bound H-atoms were placed in calculated positions (C—H 0.93–0.97 Å) and were included in the refinement in the riding model approximation, with *U*(H) set to 1.2–1.5*U*_eq_(C).

### Crystal data

**Table:**

C_17_H_23_BrN_2_O_2_	*F*(000) = 760
*M**_r_* = 367.28	*D*_x_ = 1.451 Mg m^−^^3^
Monoclinic, *P*2_1_/*n*	Mo *K*α radiation, λ = 0.71073 Å
Hall symbol: -P 2yn	Cell parameters from 5411 reflections
*a* = 7.5214 (1) Å	θ = 2.3–26.0°
*b* = 9.3693 (2) Å	µ = 2.45 mm^−^^1^
*c* = 23.8686 (5) Å	*T* = 293 K
β = 91.750 (1)°	Prism, colorless
*V* = 1681.24 (6) Å^3^	0.30 × 0.20 × 0.10 mm
*Z* = 4	

### Data collection

**Table:**

Bruker X8 APEXII diffractometer	4897 independent reflections
Radiation source: fine-focus sealed tube	3478 reflections with *I* > 2σ(*I*)
graphite	*R*_int_ = 0.045
φ and ω scans	θ_max_ = 30.0°, θ_min_ = 2.8°
Absorption correction: multi-scan (*SADABS*; Sheldrick, 1996)	*h* = −10→9
*T*_min_ = 0.526, *T*_max_ = 0.791	*k* = −13→13
25590 measured reflections	*l* = −28→33

### Refinement

**Table:**

Refinement on *F*^2^	Primary atom site location: structure-invariant direct methods
Least-squares matrix: full	Secondary atom site location: difference Fourier map
*R*[*F*^2^ > 2σ(*F*^2^)] = 0.051	Hydrogen site location: inferred from neighbouring sites
*wR*(*F*^2^) = 0.153	H-atom parameters constrained
*S* = 1.01	*w* = 1/[σ^2^(*F*_o_^2^) + (0.082*P*)^2^ + 1.8005*P*] where *P* = (*F*_o_^2^ + 2*F*_c_^2^)/3
4897 reflections	(Δ/σ)_max_ = 0.001
201 parameters	Δρ_max_ = 1.92 e Å^−^^3^
0 restraints	Δρ_min_ = −0.68 e Å^−^^3^

### Fractional atomic coordinates and isotropic or equivalent isotropic displacement parameters (Å^2^)

**Table:**

	*x*	*y*	*z*	*U*_iso_*/*U*_eq_	
Br1	1.30982 (5)	0.07856 (4)	0.564211 (15)	0.04567 (14)	
O1	0.5859 (3)	0.4826 (2)	0.28329 (9)	0.0297 (4)	
O2	0.1496 (3)	0.4797 (2)	0.35848 (10)	0.0351 (5)	
N1	0.5074 (3)	0.7166 (2)	0.27741 (9)	0.0211 (4)	
N2	0.1710 (3)	0.7124 (2)	0.33415 (9)	0.0231 (4)	
C1	0.4344 (3)	0.8410 (3)	0.30158 (10)	0.0199 (5)	
C2	0.5247 (4)	0.9710 (3)	0.29605 (11)	0.0247 (5)	
H2	0.6331	0.9729	0.2783	0.030*	
C3	0.4550 (4)	1.0961 (3)	0.31659 (12)	0.0278 (6)	
H3	0.5153	1.1818	0.3119	0.033*	
C4	0.2944 (4)	1.0944 (3)	0.34434 (12)	0.0266 (5)	
H4	0.2476	1.1784	0.3585	0.032*	
C5	0.2056 (4)	0.9665 (3)	0.35053 (11)	0.0251 (5)	
H5	0.0991	0.9651	0.3693	0.030*	
C6	0.2725 (3)	0.8394 (3)	0.32918 (10)	0.0203 (5)	
C7	0.5848 (3)	0.7301 (3)	0.22189 (11)	0.0244 (5)	
H7A	0.5875	0.6381	0.2042	0.037*	
H7B	0.7036	0.7667	0.2259	0.037*	
H7C	0.5137	0.7942	0.1992	0.037*	
C8	0.5209 (3)	0.5879 (3)	0.30474 (11)	0.0215 (5)	
C9	0.4425 (4)	0.5856 (3)	0.36297 (11)	0.0233 (5)	
H9	0.4770	0.6735	0.3827	0.028*	
C10	0.2407 (4)	0.5863 (3)	0.35245 (11)	0.0237 (5)	
C11	−0.0223 (3)	0.7209 (3)	0.32308 (12)	0.0281 (6)	
H11A	−0.0647	0.6313	0.3084	0.042*	
H11B	−0.0476	0.7951	0.2962	0.042*	
H11C	−0.0807	0.7419	0.3573	0.042*	
C12	0.5012 (4)	0.4579 (3)	0.39872 (12)	0.0291 (6)	
H12A	0.4395	0.4608	0.4338	0.035*	
H12B	0.4662	0.3709	0.3794	0.035*	
C13	0.6995 (4)	0.4532 (3)	0.41145 (14)	0.0363 (7)	
H13A	0.7388	0.5469	0.4239	0.044*	
H13B	0.7603	0.4312	0.3772	0.044*	
C14	0.7533 (4)	0.3430 (3)	0.45629 (14)	0.0372 (7)	
H14A	0.7202	0.3782	0.4927	0.045*	
H14B	0.6884	0.2551	0.4491	0.045*	
C15	0.9504 (5)	0.3118 (4)	0.45750 (15)	0.0462 (8)	
H15A	1.0143	0.4018	0.4588	0.055*	
H15B	0.9794	0.2646	0.4228	0.055*	
C16	1.0164 (4)	0.2199 (3)	0.50614 (13)	0.0360 (7)	
H16A	1.0014	0.2709	0.5411	0.043*	
H16B	0.9465	0.1331	0.5074	0.043*	
C17	1.2095 (5)	0.1827 (5)	0.50011 (14)	0.0469 (8)	
H17A	1.2768	0.2699	0.4953	0.056*	
H17B	1.2219	0.1253	0.4666	0.056*	

### Atomic displacement parameters (Å^2^)

**Table:**

	*U*^11^	*U*^22^	*U*^33^	*U*^12^	*U*^13^	*U*^23^
Br1	0.0511 (2)	0.0394 (2)	0.0454 (2)	0.00836 (15)	−0.01604 (15)	−0.00333 (14)
O1	0.0329 (10)	0.0220 (9)	0.0342 (10)	0.0060 (8)	0.0014 (8)	−0.0021 (8)
O2	0.0311 (11)	0.0260 (10)	0.0482 (13)	−0.0106 (8)	0.0014 (9)	0.0044 (9)
N1	0.0222 (10)	0.0170 (9)	0.0243 (10)	0.0011 (8)	0.0034 (8)	−0.0001 (8)
N2	0.0189 (10)	0.0223 (10)	0.0283 (11)	−0.0039 (8)	0.0017 (8)	0.0011 (8)
C1	0.0204 (11)	0.0177 (11)	0.0215 (11)	0.0023 (9)	−0.0003 (9)	0.0006 (9)
C2	0.0231 (12)	0.0213 (11)	0.0299 (13)	−0.0023 (10)	0.0051 (10)	0.0017 (10)
C3	0.0310 (14)	0.0196 (12)	0.0329 (14)	−0.0041 (10)	0.0022 (11)	0.0000 (10)
C4	0.0279 (13)	0.0209 (12)	0.0309 (13)	0.0027 (10)	−0.0004 (10)	−0.0027 (10)
C5	0.0220 (12)	0.0259 (12)	0.0276 (13)	0.0017 (10)	0.0024 (10)	−0.0006 (10)
C6	0.0187 (11)	0.0195 (11)	0.0227 (11)	−0.0019 (9)	−0.0011 (9)	0.0008 (9)
C7	0.0242 (12)	0.0257 (12)	0.0234 (12)	0.0011 (10)	0.0033 (9)	−0.0011 (10)
C8	0.0193 (11)	0.0171 (11)	0.0281 (12)	−0.0003 (9)	−0.0020 (9)	−0.0003 (9)
C9	0.0266 (12)	0.0166 (11)	0.0264 (12)	−0.0020 (9)	−0.0009 (10)	0.0013 (9)
C10	0.0258 (12)	0.0218 (12)	0.0237 (12)	−0.0042 (10)	0.0029 (9)	−0.0007 (9)
C11	0.0183 (11)	0.0328 (14)	0.0330 (14)	−0.0036 (10)	−0.0004 (10)	−0.0013 (11)
C12	0.0359 (15)	0.0201 (11)	0.0311 (14)	−0.0012 (11)	−0.0019 (11)	0.0030 (10)
C13	0.0343 (15)	0.0339 (15)	0.0403 (16)	−0.0016 (13)	−0.0050 (12)	0.0139 (13)
C14	0.0436 (17)	0.0312 (15)	0.0365 (16)	0.0045 (13)	−0.0029 (13)	0.0103 (12)
C15	0.0474 (19)	0.054 (2)	0.0369 (17)	0.0059 (17)	−0.0025 (14)	0.0178 (15)
C16	0.0428 (17)	0.0348 (15)	0.0298 (14)	−0.0013 (13)	−0.0064 (12)	0.0079 (12)
C17	0.0480 (19)	0.059 (2)	0.0334 (16)	0.0098 (17)	−0.0005 (14)	0.0101 (15)

### Geometric parameters (Å, °)

**Table:**

Br1—C17	1.947 (3)	C9—C12	1.527 (4)
O1—C8	1.221 (3)	C9—C10	1.531 (4)
O2—C10	1.222 (3)	C9—H9	0.9800
N1—C8	1.373 (3)	C11—H11A	0.9600
N1—C1	1.419 (3)	C11—H11B	0.9600
N1—C7	1.469 (3)	C11—H11C	0.9600
N2—C10	1.359 (3)	C12—C13	1.514 (4)
N2—C6	1.420 (3)	C12—H12A	0.9700
N2—C11	1.472 (3)	C12—H12B	0.9700
C1—C6	1.402 (3)	C13—C14	1.533 (4)
C1—C2	1.402 (4)	C13—H13A	0.9700
C2—C3	1.380 (4)	C13—H13B	0.9700
C2—H2	0.9300	C14—C15	1.511 (5)
C3—C4	1.396 (4)	C14—H14A	0.9700
C3—H3	0.9300	C14—H14B	0.9700
C4—C5	1.382 (4)	C15—C16	1.517 (4)
C4—H4	0.9300	C15—H15A	0.9700
C5—C6	1.395 (4)	C15—H15B	0.9700
C5—H5	0.9300	C16—C17	1.505 (5)
C7—H7A	0.9600	C16—H16A	0.9700
C7—H7B	0.9600	C16—H16B	0.9700
C7—H7C	0.9600	C17—H17A	0.9700
C8—C9	1.526 (4)	C17—H17B	0.9700
			
C8—N1—C1	123.5 (2)	N2—C11—H11A	109.5
C8—N1—C7	118.6 (2)	N2—C11—H11B	109.5
C1—N1—C7	117.7 (2)	H11A—C11—H11B	109.5
C10—N2—C6	123.5 (2)	N2—C11—H11C	109.5
C10—N2—C11	118.3 (2)	H11A—C11—H11C	109.5
C6—N2—C11	118.0 (2)	H11B—C11—H11C	109.5
C6—C1—C2	118.9 (2)	C13—C12—C9	113.7 (2)
C6—C1—N1	122.3 (2)	C13—C12—H12A	108.8
C2—C1—N1	118.7 (2)	C9—C12—H12A	108.8
C3—C2—C1	120.9 (2)	C13—C12—H12B	108.8
C3—C2—H2	119.5	C9—C12—H12B	108.8
C1—C2—H2	119.5	H12A—C12—H12B	107.7
C2—C3—C4	120.2 (2)	C12—C13—C14	113.4 (3)
C2—C3—H3	119.9	C12—C13—H13A	108.9
C4—C3—H3	119.9	C14—C13—H13A	108.9
C5—C4—C3	119.3 (2)	C12—C13—H13B	108.9
C5—C4—H4	120.4	C14—C13—H13B	108.9
C3—C4—H4	120.4	H13A—C13—H13B	107.7
C4—C5—C6	121.4 (2)	C15—C14—C13	112.4 (3)
C4—C5—H5	119.3	C15—C14—H14A	109.1
C6—C5—H5	119.3	C13—C14—H14A	109.1
C5—C6—C1	119.4 (2)	C15—C14—H14B	109.1
C5—C6—N2	118.9 (2)	C13—C14—H14B	109.1
C1—C6—N2	121.7 (2)	H14A—C14—H14B	107.9
N1—C7—H7A	109.5	C14—C15—C16	115.0 (3)
N1—C7—H7B	109.5	C14—C15—H15A	108.5
H7A—C7—H7B	109.5	C16—C15—H15A	108.5
N1—C7—H7C	109.5	C14—C15—H15B	108.5
H7A—C7—H7C	109.5	C16—C15—H15B	108.5
H7B—C7—H7C	109.5	H15A—C15—H15B	107.5
O1—C8—N1	122.4 (2)	C17—C16—C15	110.6 (3)
O1—C8—C9	122.8 (2)	C17—C16—H16A	109.5
N1—C8—C9	114.8 (2)	C15—C16—H16A	109.5
C12—C9—C8	114.0 (2)	C17—C16—H16B	109.5
C12—C9—C10	111.3 (2)	C15—C16—H16B	109.5
C8—C9—C10	105.0 (2)	H16A—C16—H16B	108.1
C12—C9—H9	108.8	C16—C17—Br1	113.1 (2)
C8—C9—H9	108.8	C16—C17—H17A	109.0
C10—C9—H9	108.8	Br1—C17—H17A	109.0
O2—C10—N2	122.4 (3)	C16—C17—H17B	109.0
O2—C10—C9	122.3 (2)	Br1—C17—H17B	109.0
N2—C10—C9	115.3 (2)	H17A—C17—H17B	107.8
			
C8—N1—C1—C6	−47.6 (3)	C1—N1—C8—C9	2.9 (3)
C7—N1—C1—C6	137.3 (2)	C7—N1—C8—C9	177.9 (2)
C8—N1—C1—C2	134.5 (3)	O1—C8—C9—C12	17.7 (4)
C7—N1—C1—C2	−40.6 (3)	N1—C8—C9—C12	−164.4 (2)
C6—C1—C2—C3	−1.0 (4)	O1—C8—C9—C10	−104.4 (3)
N1—C1—C2—C3	177.0 (2)	N1—C8—C9—C10	73.5 (3)
C1—C2—C3—C4	1.3 (4)	C6—N2—C10—O2	177.1 (3)
C2—C3—C4—C5	−0.6 (4)	C11—N2—C10—O2	2.0 (4)
C3—C4—C5—C6	−0.6 (4)	C6—N2—C10—C9	−5.0 (4)
C4—C5—C6—C1	0.9 (4)	C11—N2—C10—C9	179.9 (2)
C4—C5—C6—N2	−176.7 (2)	C12—C9—C10—O2	−18.6 (4)
C2—C1—C6—C5	−0.2 (4)	C8—C9—C10—O2	105.2 (3)
N1—C1—C6—C5	−178.1 (2)	C12—C9—C10—N2	163.5 (2)
C2—C1—C6—N2	177.4 (2)	C8—C9—C10—N2	−72.7 (3)
N1—C1—C6—N2	−0.5 (4)	C8—C9—C12—C13	62.5 (3)
C10—N2—C6—C5	−133.0 (3)	C10—C9—C12—C13	−178.9 (2)
C11—N2—C6—C5	42.1 (3)	C9—C12—C13—C14	168.8 (3)
C10—N2—C6—C1	49.5 (4)	C12—C13—C14—C15	164.8 (3)
C11—N2—C6—C1	−135.5 (3)	C13—C14—C15—C16	171.7 (3)
C1—N1—C8—O1	−179.2 (2)	C14—C15—C16—C17	174.1 (3)
C7—N1—C8—O1	−4.1 (4)	C15—C16—C17—Br1	175.0 (3)

### Hydrogen-bond geometry (Å, °)

**Table:**

*D*—H···*A*	*D*—H	H···*A*	*D*···*A*	*D*—H···*A*
C7—H7B···O1^i^	0.96	2.58	3.430 (3)	147
C7—H7C···O2^ii^	0.96	2.51	3.471 (3)	174
C11—H11B···O1^ii^	0.96	2.60	3.551 (3)	173

Symmetry codes: (i) −*x*+3/2, *y*+1/2, −*z*+1/2; (ii) −*x*+1/2, *y*+1/2, −*z*+1/2.
